# Characteristics of macrophage aggregates prepared by rotation culture and their response to polymeric materials

**DOI:** 10.1007/s10047-023-01428-6

**Published:** 2024-01-09

**Authors:** Shota Toda, Yoshihide Hashimoto, Naoko Nakamura, Masahiro Yamada, Ryusuke Nakaoka, Wataru Nomura, Masaya Yamamoto, Tsuyoshi Kimura, Akio Kishida

**Affiliations:** 1 Institute of Biomaterials and Bioengineering, Tokyo Medical and Dental University, 2-3-10 Kanda-Surugadai, Chiyoda-ku, Tokyo 101-0062 Japan; 2 Department of Bioscience and Engineering, Shibaura Institute of Technology, 307 Fukasaku, Minuma-ku, Saitama-shi, Saitama 337-8570 Japan; 3 Division of Molecular and Regenerative Prosthodontics, Graduate School of Dentistry, Tohoku University, 4-1 Seiryo-machi, Aoba-ku, Sendai 980-8575 Japan; 4 Division of Medical Devices, National Institute of Health Sciences, 3-25-26 Tonomachi, Kawasaki-ku, Kawasaki-shi, Kanagawa 210-9501 Japan; 5 Graduate School of Biomedical and Health Sciences, Hiroshima University, 1-2-3 Kasumi, Minami-ku, Hiroshima-shi, Hiroshima 734-8553 Japan; 6 Department of Materials Processing, Graduate School of Engineering, Tohoku University, 6-6-02 Aramaki-aza Aoba, Aoba-ku, Sendai 980-8579 Japan

**Keywords:** THP-1 cell, Rotation culture, Macrophage aggregate, Material property evaluation

## Abstract

Understanding the interaction between macrophages and biomaterials is important for the creation of new biomaterials and the development of technologies to control macrophage function. Since macrophages are strongly adhesive, caution is required when performing in vitro evaluations. Similarly, when THP-1 cells, macrophage precursor cells, are differentiated into macrophages using phorbol-12-myristate-13-acetate (PMA), it becomes difficult to detach them from the adherent substrate, which has been a problem on investigation of immunological responses to biomaterials. In this study, the interaction of THP-1 cell-differentiated macrophages with biomaterials was analyzed based on a new method of seeding THP-1 cells. THP-1 cells were cultured in static and rotation culture without and with PMA. In undifferentiated THP-1 cells, there was no change in cellular function between static and rotation cultures. In rotation culture with PMA, THP-1 cells differentiated and formed macrophage aggregates. IL-1β and MRC1 expression in macrophage aggregates was examined after differentiation and M1/M2 polarization. Macrophage aggregates in rotation culture tended to be polarized toward M2 macrophages compared with those in static culture. In the evaluation of the responses of macrophage aggregates to several kinds of polymeric materials, macrophage aggregates showed different changes in MRC1 expression over time at 30, 50, and 70 rpm. Rotation speed of 30 rpm was considered most appropriate condition in that it gave stable results with the same trend as obtained with static culture. The use of macrophage aggregates obtained by rotational culture is expected to provide new insights into the evaluation of inflammatory properties of biomaterials.

**Supplementary Information:**

The online version contains supplementary material available at 10.1007/s10047-023-01428-6.

## Introduction

Inflammatory reactions are elicited when biomaterials are implanted in vivo, and biomaterials are encapsulated by a fibrous capsule at the end phase of inflammatory reactions. Encapsulation reactions can decrease the in vivo stability of biomaterials and should be reduced as much as possible. However, it is difficult to predict the encapsulation of biomaterials in vitro, and animal studies are considered necessary. On the other hand, the initial inflammatory reaction is known to be related to the subsequent fibrotic response [1]. If the initial inflammatory reaction in vivo can be predicted, this would aid the understanding of the inflammatory reactions of biomaterials and it would lead the development of new implantable materials. As a result, the need for animal testing is expected to decrease. Early inflammation is mainly regulated by immune cells, although its intensity is determined by various factors. Macrophages play a pivotal role in this, and the interaction between macrophages and biomaterials in vitro has been studied [2]. Recent research has shown that, under inflammatory conditions, M0 macrophages (M0) differentiate from monocytes, migrate to tissues, and are polarized into inflammatory macrophages (M1) in early inflammation and into healing macrophages (M2) in late inflammation. The evaluation of such differentiation and polarization in vitro should deepen our understanding of the inflammatory properties of biomaterials.

The primary culture of macrophages is expected to provide substantial information. However, their preparation is complicated, requires expertise, and is difficult to reproduce. Therefore, THP-1 cells (THP-1) have attracted attention as macrophage progenitor cells in the established cell line; these cells are easy to subculture and usually differentiate into macrophages upon exposure to phorbol-12-myristate-13-acetate (PMA). Because macrophages derived from THP-1 can recognize foreign substances after PMA-induced differentiation [3], THP-1 are widely used in macrophage research as a monocytic cell model [4]. A common experimental method is to seed dispersed THP-1 on materials in static culture and add PMA to induce their differentiation into macrophages, followed by evaluation of whether M1 or M2 polarization occurs [5]. THP-1 stimulated with PMA are highly adherent to substrates and are difficult to detach and use in subsequent seeding experiments [6]. Therefore, when THP-1 are used for material evaluation, macrophages are induced to differentiate with PMA after seeding. However, in this method, macrophages interact with materials in a manner that differs from that in vivo. Generally, in vivo, after receiving inflammatory signals, monocytes extravasate, differentiate into macrophages, and migrate to inflammatory sites [7]. When material is implanted, inflammatory reactions occur. Given that macrophages are one type of cell that regulates the inflammatory response, it is important to understand the functional changes that occur when macrophages are in direct contact with such material. It is thus necessary to devise a method for seeding THP-1 and differentiated macrophages. We investigated the timing of PMA addition to THP-1 and found that its addition in a suspended state leads to the formation of aggregates. If the cells in these aggregates are macrophages, this approach could potentially be applied to evaluate their interaction with biomaterials. We investigated the formation of aggregates when THP-1 differentiate into macrophages using a rotation culture method, and examined the possibility of applying this method to immunological evaluation of biomaterials.

## Materials and methods

### Materials

Spectra/Por^®^ 6 Dialysis Membrane (MWCO: 10 kDa, Spectrum Laboratories, CA, USA) was purchased and used as cellulose. We obtained 6-nylon (KOKUGO, Tokyo, Japan), polyethylene terephthalate film (PET, Toray Industry, Tokyo, Japan), polytetrafluoroethylene (PTFE, Fron Industry, Tokyo, Japan), polymethylmethacrylate (PMMA, MW 350000, Sigma, St. Louis, MO, USA) and 24-well tissue culture plates (TCPS, IWAKI, Shizuoka, Japan). CELLSPIN and 100 ml spinner flasks were purchased from Pfeiffer Electronic Engineering GmbH (Lahnau, Germany). PMA, RPMI-1640 with L-glutamine and lipopolysaccharide from Escherichia coli O26 (LPS) were purchased from FUJIFILM Wako (Osaka, Japan). FBS was purchased from Biowest (Nuaillé, France). Recombinant human IFN-g, IL-4 and IL-13 were obtained from BioLegend (CA, USA). ISOGEN with a spin column was purchased from NipponGene (Tokyo, Japan). The ReverTra Ace qPCR RT Master Mix and THUNDERBIRD™ SYBR^®^ qPCR Mix were obtained from Toyobo (Osaka, Japan). THP-1 were purchased from JCRB Cell Bank (Osaka, Japan).

### Preparation of polymeric materials

Cellulose was immersed in distilled water and cut into 15-mm-diameter discs. PMMA film was obtained by a casting method. Nylon, PET, and PTFE were also cut into 15-mm-diameter discs, extracted in a Soxhlet apparatus and then immersed in distilled water.

### Cell seeding experiments

**Fig. 1:**
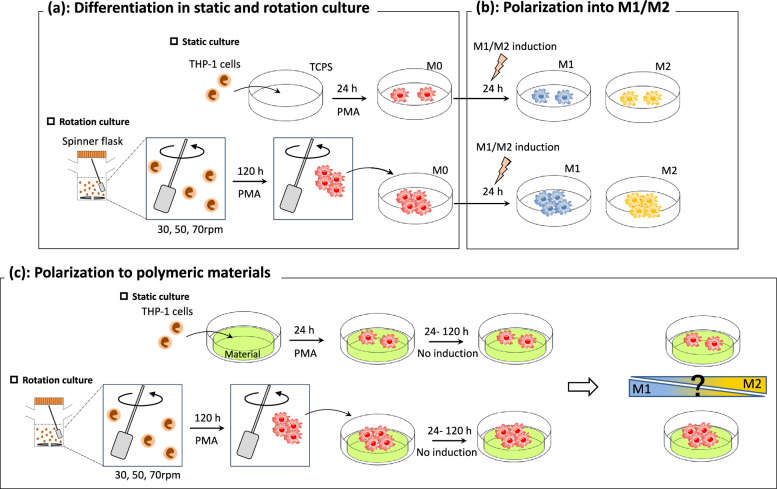
Schematic illustrations of this study. **a** Differentiation in static and rotation culture, **b** polarization into M1/M2 and **c** polarization to polymeric materials

The following cell seeding experiments were performed, as shown in Fig. 1. Experiments of differentiation in static and rotation culture, polarization into M1/M2 and polarization to polymeric materials were shown in Fig. 1a–c, respectively.

### Differentiation in static and rotation culture

THP-1 were cultured in RPMI-1640 containing 10% FBS and 1% penicillin/streptomycin. For differentiation in static culture, THP-1 at 4 × 10^5^ cells/well were differentiated by incubation for 24 h with 320 nM PMA on TCPS. For differentiation in rotation culture, THP-1 at 1 × 10^7^ cells were differentiated by incubation for 120 h with 320 nM PMA in spinner flasks (30, 50, or 70 rpm). As controls, THP-1 were cultured for the same periods in the absence of PMA. After differentiation, macrophages were stained with Calcein AM and PI and cells were observed using a fluorescence microscope (Keyence Corp., Osaka, Japan).

### Polarization into M1/M2

After differentiation in static or rotation culture, polarization of the macrophages was induced. In static culture, PMA-containing medium was removed, and medium containing 20 ng/ml IFN- γ and 50 ng/ml LPS (M1 induction), 20 ng/ml IL-4 and 20 ng/ml IL-13 (M2 induction), or without PMA (No induction) was added. Macrophages in these three different media were cultured for 24 h in static culture. Meanwhile, in rotation culture, aggregated macrophages were retrieved, centrifuged to remove PMA-containing medium, and the same three types of media as in static culture were added. Then, macrophage aggregates together with the three different media were re-seeded on TCPS and further incubated for 24 h for M1/M2 polarization in static culture.

### Macrophage culture on various polymeric materials

Prior to seeding, the disk-shaped materials were placed onto TCPS, and stainless-steel rings were placed onto the materials. In static culture, THP-1 were seeded at 4 × 10^5^ cells/well on the materials and cultured for 24 h in PMA-containing medium. Then, the PMA-containing medium was exchanged for PMA-free medium, followed by culture of the cells for 24, 72, or 120 h on each material. Meanwhile, in rotation culture, aggregated macrophages were obtained by the same differentiation procedure as mentioned above. After changing the medium, aggregated macrophages with PMA-free medium were re-seeded and cultured for 24, 72, or 120 h on the materials.

### Measurement of aggregate size

Aggregated macrophages were randomly photographed under a microscope after differentiation in rotation culture and the size of the aggregates was analyzed using ImageJ software.

### Real-time polymerase chain reaction (PCR)

RNA was extracted from cells on materials using ISOGEN with a spin column. cDNA was synthesized using ReverTra Ace™ qPCR RT Master Mix and RT-PCR was performed using THUNDERBIRD™ SYBR^®^ qPCR Mix (95 °C for 60 s, 95 °C for 15 s and 60 °C for 60 s, for 40 cycles). The results were analyzed using the 2^-ΔΔCt^ method. The primers used were as follows:

**Table:** 

IL-1β	(forward)	5′-ATGATGGCTTATTACAGTGGCAA-3’
	(reverse)	5’-GTCGGAGATTCGTAGCTGGA-3′
MRC1	(forward)	5′-CTACAAGGGATCGGGTTTATGGA-3′
	(reverse)	5′-TTGGCATTGCCTAGTAGCGTA-3′
ACTβ	(forward)	5′-CATGTACGTTGCTATCCAGGC-3′
	(reverse)	5′-CTCCTTAATGTCACGCACGAT-3′

### Statistical analysis

Each experiment was performed at least three times. The results are expressed as mean ± standard deviation. One-way analysis of variance (ANOVA) and Tukey’s post hoc multiple comparison tests were carried out to evaluate statistical significance. A *p*-value < 0.05 was considered statistically significant.

## Results

### Morphological appearance of THP-1 and macrophages

**Fig. 2:**
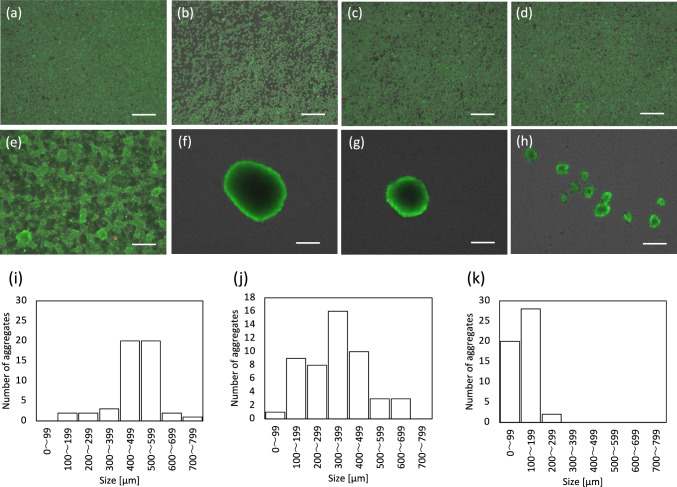
Fluorescent images of THP-1 and macrophages in static and rotation culture. **A**–**d** PMA(–), **e–h** PMA(+). **a** THP-1 in static culture, **b** THP-1 in 30 rpm rotation culture, **c** THP-1 in 50 rpm rotation culture, **d** THP-1 in 70 rpm rotation culture, **e** M0 macrophages in static culture, **f** aggregated macrophages in 30 rpm rotation culture, **g** aggregated macrophages in 50 rpm rotation culture, and **h** aggregated macrophages in 70 rpm rotation culture. Scale bar: 200 µm. Histograms of aggregated macrophages in rotation culture. **i** 30 rpm, **j** 50 rpm, **k** 70 rpm

The results of static and rotation culture of THP-1 for 120 h are shown in Fig. 2. THP-1 can be passaged in static culture without PMA. During passaging, THP-1 seeded on TCPS and cultured statically showed proliferative activity but not adhesion to the substrate. THP-1 in this state are considered to be undifferentiated (Fig. 2a). When THP-1 were cultured in a spinner flask, they proliferated as in static culture and were spherical in shape and dispersed (Fig. 2b–d). Meanwhile, when PMA was added, THP-1 differentiated into macrophages. To ensure macrophage differentiation, 320 nM PMA was added, and the cells were cultured for more than 24 h [8]. In the control culture on TCPS, THP-1 were seeded with PMA in a dispersion solution and differentiated into macrophages. Figure 2f–h shows the results of rotation culture at 120 h after PMA addition. Aggregates were observed at all rotation speeds from 30 to 70 rpm, indicating the strong aggregation ability of macrophages.

Since THP-1 did not aggregate when PMA was not added, there was no effect of rotation culture on the differentiation of THP-1 into macrophages under these conditions. This indicates that PMA stimulates the differentiation of THP-1 into macrophages, leading to the formation of aggregates. As for the incubation time, macrophage aggregation was observed after 24 h of rotation culture, but there were many cells that did not aggregate and the aggregate formation was nonuniform, so the incubation time was extended up to 120 h (Fig. S1). The acquisition of adhesiveness associated with differentiation into macrophages was observed at 24 h in TCPS, but in the case of rotation culture, stable aggregate formation required more time.

Figure 2i–k shows histograms of macrophage aggregate size after 120 h of rotation culture. 24 h after the addition of PMA, only small aggregates of less than 100 µm were observed in the suspension, but as the rotation culture period was extended, aggregates of uniform size were formed. Specifically, the size of aggregates was 400–599 µm at 30 rpm, 300–399 µm at 50 rpm, and 100–199 µm at 70 rpm, showing a decrease as the rotation speed increased. This may have been due to shear stress in rotation culture.

### Gene expression of THP-1 and macrophages prepared in static and rotation culture after differentiation and polarization

**Fig. 3:**
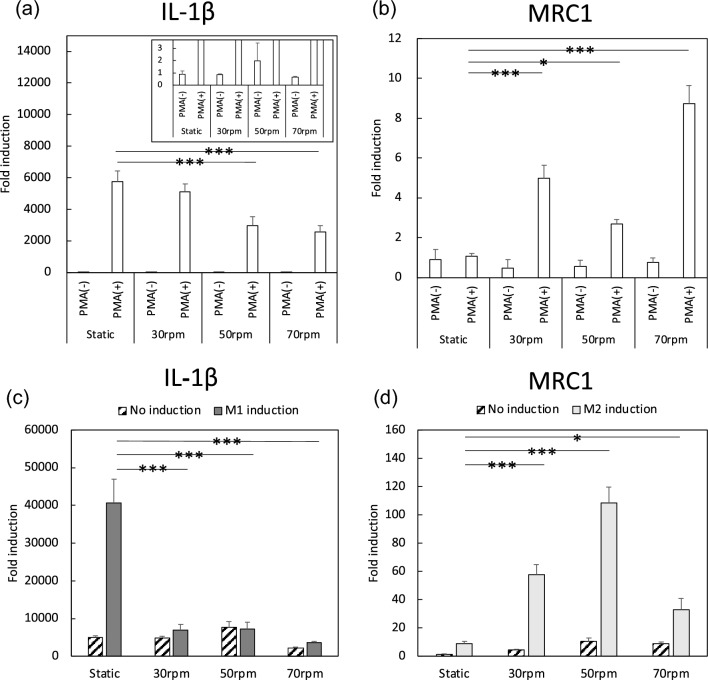
**a** IL-1β and **b** MRC1 gene expression of macrophages after differentiation in static and rotation culture. **c** IL-1β and **d** MRC1 gene expression of M1/M2 polarized macrophages after differentiation in static and rotation culture. *p < 0.05, ***p < 0.001

The differentiated and undifferentiated states of THP-1 were evaluated by IL-1β and MRC1 expression in Fig. 3a, b. Compared with those in THP-1 in static culture, IL-1β and MRC1 expression in THP-1 in rotation culture with PMA(−) were unchanged: 30 rpm (IL-1β: 0.84-fold, MRC1: 0.48-fold), 50 rpm (IL-1β: 1.98-fold, MRC1: 0.56-fold), and 70 rpm (IL-1β: 0.64-fold, MRC1: 0.74-fold). These results indicate that THP-1 remained undifferentiated when cultured under PMA(−) rotation conditions. Meanwhile, when THP-1 underwent PMA(+) rotation culture, decreases in IL-1β were observed at 50 and 70 rpm (50 rpm: 2960-fold, 70 rpm: 2581-fold), and increases were found in MRC1 mRNA at 30, 50, and 70 rpm (30 rpm: 4.99-fold, 50 rpm: 2.67-fold, 70 rpm: 8.74-fold). These results indicate that THP-1 can maintain an undifferentiated state in both static and rotation cultures without PMA. IL-1β expression was lower, while MRC1 expression was higher in rotation cultures. This indicates that macrophage aggregates in rotation cultures tend to polarize toward M2.

Since the macrophage aggregates obtained in rotation culture were considered to polarize to M1 or M2, we examined their polarization ability using polarizing agents. Figure 3c, d shows IL-1β and MRC1 expression in the presence of LPS/IFN-γ (M1 induction) and IL-4/IL-13 (M2 induction). In static culture, IL-1β was increased by M1 induction compared to that by No induction, while IL-1β expression in rotation culture did not increase (Fig. 3c). Meanwhile, as shown in Fig. 3d, MRC1 expression upon M2 induction was ninefold in static culture, 56-fold at 30 rpm, 109-fold at 50 rpm, and 33-fold at 70 rpm compared with the levels without such induction. These results showed that M2 induction tended to increase MRC1 expression in macrophage aggregates prepared in rotation culture, suggesting that macrophage aggregates prepared in rotation culture are less sensitive to M1 induction but more sensitive to M2 induction than macrophages prepared in static culture.

### Gene expression of macrophage aggregates on various biomaterials

**Fig. 4:**
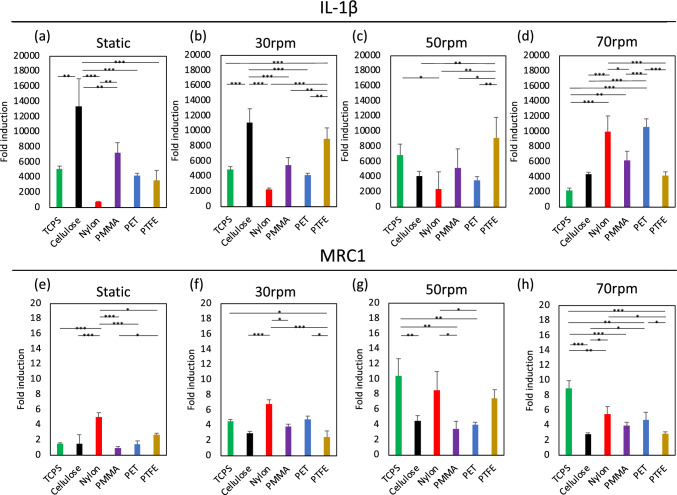
IL-1β and MRC1 gene expression of macrophages differentiated in static and rotation culture upon 24 h of exposure to polymeric materials. **a, e** Static culture, **b, f** 30 rpm rotation culture, **c, g** 50 rpm rotation culture, **d, h** 70 rpm rotation culture. *p < 0.05, **p < 0.01, ***p < 0.001

**Fig. 5:**
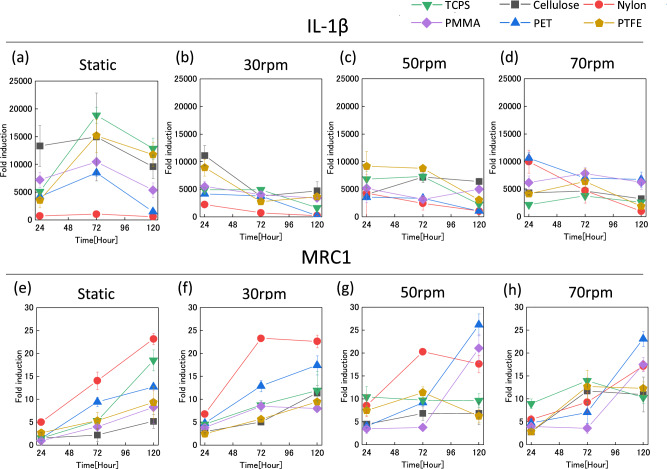
Time course of IL-1β and MRC1 gene expression of macrophages differentiated in static and rotation culture upon exposure to polymeric materials. **a, e** Static culture, **b, f** 30 rpm rotation culture, **c, g** 50 rpm rotation culture, **d, h** 70 rpm rotation culture

The interaction between macrophage aggregates obtained in rotation culture and polymeric materials was investigated. Figure 4 shows IL-1β and MRC1 expression of macrophages after 24 h of incubation on polymeric materials. At 24 h, IL-1β expression of macrophages clearly differed among the polymeric materials. In static culture and upon rotation at 30 rpm, IL-1β expression of macrophages on Cellulose was highest, but this trend was not observed at 50 and 70 rpm. Meanwhile, MRC1 expression at 50 and 70 rpm was a little higher than that in static culture and at 30 rpm. These results indicate that macrophages differentiated in each condition may have differently recognized the material properties to an extent dependent on their differentiation stage. Figure 5 shows the changes in IL-1β and MRC1 expression of macrophages on materials over time. For IL-1β expression in static culture, there was an increase in expression at 72 h and a slight decrease at 120 h for all samples (Fig. 5a). For IL-1β expression in rotation culture, it showed a generally decreasing trend from 24 to 120 h, and the expression levels were also lower than those in static cultures (Fig. 5b–d). Meanwhile, MRC1 expression tended to increase over time in all samples (Fig. 5e–h). Samples for which unexpected behavior was exhibited were PET and PMMA. Macrophages on these samples showed low MRC1 expression in static culture, but increased expression in rotation culture at 50 and 70 rpm.

## Discussion

Many studies involving the immunological evaluation of materials using macrophage polarization as an indicator have been reported. In this study, THP-1 were differentiated into macrophages using rotation culture methods to form aggregates, and the differentiation and polarization characteristics of the macrophage formed aggregates were examined. Then, the possibility of using macrophage aggregates to immunologically evaluate the properties of biomaterials was investigated.

We also examined the effect of rotation speed and found that THP-1 cultured in rotation culture without PMA maintained a dispersed state at all rotation speeds, with no aggregate formation observed. In contrast, as shown in Fig. 2f–h, THP-1 in rotation culture with PMA formed aggregates. The aggregate size was larger at 30 rpm and smaller at 70 rpm (Fig. 2i–k). This trend was similar to that reported previously [9], which was thought to be due to the effect of shear stress during rotation culture. In static culture, THP-1 showed strong adhesion to the substrate 24 h after the addition of PMA, but in rotation culture, they took 120 h to form stable aggregates. This may have been due to cell–cell interactions for aggregation differing from the mechanism of adhesion to the substrate. However, the details of this are unknown and require further investigation.

The effects of rotation culture on the gene expression of THP-1 and macrophages were examined. In PMA(−) conditions, the expression of IL-1β and MRC1 was almost the same after static and rotation culture, suggesting that rotation culture had a negligible effect. In our previous report, we described that contact between THP-1 and materials does not trigger differentiation into macrophages. Given that there was no effect on differentiation in this rotation culture, THP-1 is considered to be less sensitive to the culture environment.

It has been reported that various receptors are expressed during differentiation into macrophages, suggesting that they recognize the fluid in rotation culture. Macrophages aggregated in rotation culture show increased MRC1 expression, indicating polarization toward M2-like macrophages. Furthermore, in terms of the responses of macrophages to subsequent stimulation with polarizing agents, responses to M1 induction were inhibited but those to M2 induction were promoted (Fig. 3). These results suggest that macrophage aggregates obtained in rotation culture are susceptible to M2 polarization. Other researchers have reported that, when macrophages are cultured in the presence of medium flow, the gene expression profile represents an M2-like state [10], suggesting that physical stimuli control the activation state of macrophages. Macrophage aggregates in rotation culture showed the same gene expression trend, supporting the assertion that rotation culture induces M2 polarization. When macrophages differentiated in static culture are subjected to M2 induction, MRC1 expression is low compared with IL-1β in M1 induction. Since macrophage aggregates under the M2 induction show higher MRC1 expression with rotation culture than with static culture, rotation culture can be considered as a method for selectively inducing M2 macrophages. It is anticipated that this approach could provide a new macrophage induction method for the study of inflammatory responses.

Next, we discuss the macrophage aggregates that can be prepared in rotation culture. At the final stage of the inflammatory process in vivo, macrophages fuse to become foreign body giant cells (FBGCs). According to some reports, their characteristics have been revealed [11]. When the macrophage aggregates were exposed to trypsin to abolish intercellular adhesion, the macrophages adopted a dispersed state. This suggests that the macrophage aggregates were possible precursors of FBGCs. Since long-term rotation culture of macrophages is difficult, it was not possible to confirm whether the aggregates in this study changed into an FBGC-like form. As such, future studies on this issue are needed. In general, cell aggregates are reported to express functions different from those of dispersed cells in static culture because they exhibit active cell–cell communication [12]. Regarding macrophages, it has been reported that the formation of macrophage aggregates in 3D scaffold culture was associated with lower angiogenic ability [13]. The macrophage aggregates are also thought to have undergone changes in cellular function due to aggregation.

The Interaction of macrophage aggregates prepared by rotation culture with polymeric materials was also evaluated. Figure 4 shows the results of gene expression 24 h after seeding. MRC1 expression was upregulated in rotation culture. The variation in such expression was small and stable at 30 rpm, while the variation tended to be larger at 50 and 70 rpm. Based on these results, we considered 30 rpm to be appropriate when macrophage aggregates are used for the immunological evaluation of biomaterials. Under these conditions, IL-1β expression was inhibited and MRC1 expression was promoted. 50 and 70 rpm may cause functional changes of cells in the state of aggregation in rotation culture, in addition to stimulation from the material on which they are located. Therefore, further investigation is necessary to understand the macrophage function after rotation culture.

Figure 5 shows the temporal changes in gene expression. Overall, IL-1β expression was high in static culture and low in rotation culture. MRC1 expression of macrophages after 120 h of contact with PET and PMMA was low in static culture, while it was high upon rotation culture at 50 and 70 rpm. Some studies have reported that PMMA and PET induced a fibrous membrane [14, 15]. These results suggest that the response of dispersed macrophages prepared in static culture to material reflects the early stage of a foreign body reaction, which is comparatively M1-dominant, while that of aggregated macrophages prepared in rotation culture reflects the later stage of a foreign body reaction, which is M2-dominant. The results also suggest that 30 rpm is an appropriate setting for rotation culture for the immunological evaluation of biomaterials for the same reason as in Fig. 4.

## Conclusion

We prepared macrophage aggregates by optimizing the rotation speed in rotation culture. With respect to cellular morphology, increasing rotation speeds were associated with smaller aggregates. Regarding macrophage function, macrophage aggregates in rotation culture expressed the M2 gene compared with those in static culture, while their response to M1 induction was suppressed and that to M2 induction was promoted. This suggested that rotation culture induces polarization to the M2 phenotype. For applications in biomaterial evaluation, 30 rpm can be considered the most appropriate rotation setting in that it produces the same trend of results as static culture. These results provide useful insights into the fundamental properties of macrophages and the immunological evaluation of biomaterials.

## Supplementary Information

Below is the link to the electronic supplementary material.

Supplementary file 1 (PDF 98 KB)

## Data Availability

The data underlying this article will be shared on reasonable request to the corresponding author.
