# Supplementary material for: Characteristics of macrophage aggregates prepared by rotation culture and their response to polymeric materials
Source: J Artif Organs. 2024 Jan 9;27(4):410–8. doi: 10.1007/s10047-023-01428-6 (PMC11582132; doi:10.1007/s10047-023-01428-6)
Supplement: Supplementary file 1 — (PDF 98 KB) [file 10047_2023_1428_MOESM1_ESM.pdf]

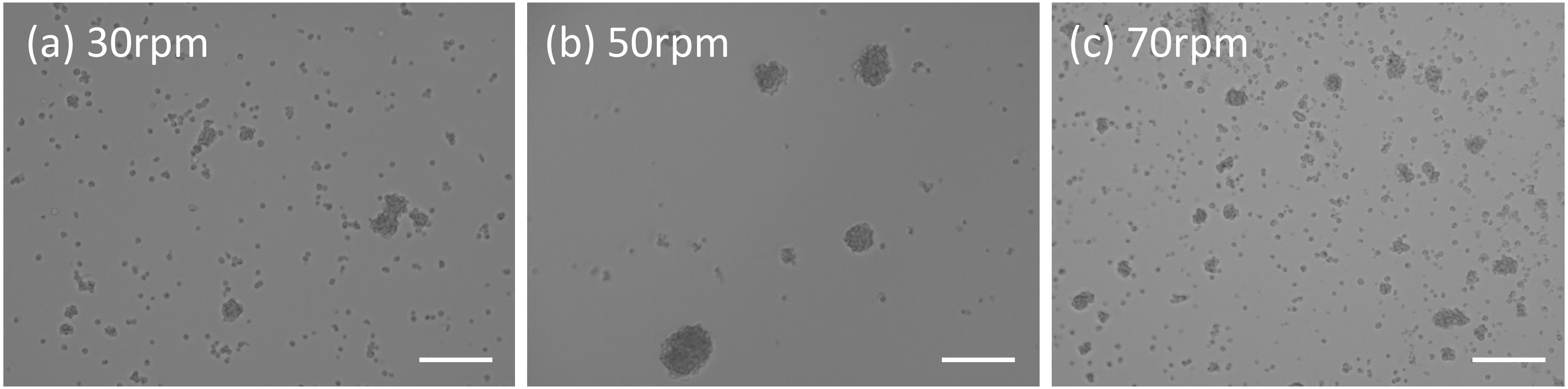

Fig. S1 Phase-contrast images of macrophages in rotation culture for 24 h. (a) 30 rpm, (b) 50 rpm, (c) 70 rpm. Scale bar: 200 µm.
